# A review of risk factors associated with insulin omission for weight loss in type 1 diabetes

**DOI:** 10.1177/13591045211026142

**Published:** 2021-06-13

**Authors:** Rebecca Hall, Leanna Keeble, Sandra-Ilona Sünram-Lea, Michelle To

**Affiliations:** Department of Psychology, 4396 Lancaster University, Lancaster, UK

**Keywords:** Type 1 diabetes, diabulimia, insulin omission, gender, mental health, anxiety, depression, weight concern, body dissatisfaction, eating disorder

## Abstract

Research suggests that as many as 60% of people with type 1 diabetes (T1D) admit to misusing insulin. Insulin omission (IO) for the purpose of weight loss, often referred to as diabulimia, is a behaviour becoming increasingly recognised, not least since prolonged engagement can lead to serious vascular complications and mortality. Several risk factors appear to be relevant to the development of IO, most notably gender, anxiety and depression and increased weight concerns and body dissatisfaction. Evidence suggests that women, especially young girls, are more likely to omit insulin as a method of weight loss compared to men. Mental health conditions such as anxiety and depression are increasingly prevalent in people with T1D compared to their peers, and appear to contribute to the risk of IO. Increased weight concerns and body dissatisfaction are further prominent risk factors, especially given increases in weight which often occur following diagnosis and the monitoring of weight by diabetes teams. This review presents evidence examining these risk factors which increase the likelihood of a person with T1D engaging in IO and highlights the complications associated with prolongment of the behaviour. Further research looking at the comorbidities of these risk factors, alongside other factors, would provide greater insight into understanding IO in people with T1D.

## Introduction

Type 1 diabetes (T1D) is a condition which affects approximately 400,000 people in the United Kingdom alone, including 30,000 children (Diabetes UK, 2016). Type 1 diabetes is an autoimmune disease resulting in the destruction of insulin secreting β-cells within the pancreas (Todd, 2010). Insulin is no longer available to enable the cellular removal of glucose from the bloodstream, leading to rising glucose levels and resulting in hyperglycaemia (high blood sugar).

As a consequence of hyperglycaemia, profound changes in energy metabolism occur, resulting in a catabolic condition with “severe depletion of both energy stores and protein mass” (Hebert & Nair, 2010). This insulin-deficient state leads to weight loss, as the body is forced to breakdown fatty acids to maintain normal muscle and other tissue functions. This breakdown of fatty acids leads to the unregulated accumulation of ketones (an acidic by-product of fatty acid breakdown) in the bloodstream. If maintained for even a relatively short period of time (even as little as a few days), high ketone levels can lead to a highly critical state known as diabetic ketoacidosis (Misra & Oliver, 2015). Diabetic ketoacidosis can be lethal, with mortality rates of up to 30% among those admitted to hospital (Oschatz et al., 1999); furthermore, these rates increase even further amongst young children (Edge et al., 1999).

Insulin omission (IO) is a behaviour engaged in by people with T1D. Research suggests that it may be as a method of weight loss (Polonsky et al., 1994). Short-term symptoms of IO are those of hyperglycaemia: excessively high blood glucose (>11%), increased ketone levels, thirst, frequent urination, fatigue and cognitive deficits such as lack of concentration (Ruth-Sahd et al., 2009). The longer and more frequent the IO, and the longer the hyperglycaemic state persists, the more severe the symptoms: weight loss, severe dehydration and increased glycosylated haemoglobin (HbA1c). HbA1c relates to the average plasma glucose concentration over a three-month period and is therefore a good indicator of blood glucose levels within that period. Persistent IO, therefore, leads to prolonged periods of hyperglycaemia and increased HbA1c, which can lead to the increased development of a number of vascular complications (Calcutt et al., 2009) and increased risk of mortality (Goebel-Fabbri et al., 2008).

Prevalence rates of IO vary widely depending on the methodology used, but studies using the Diabetes Eating Problem Survey-Revised (DEPS-R; Markowitz et al., 2010), a questionnaire considered one of the most psychometrically robust methods currently available in assessing insulin manipulation, have reported prevalence as high as 60.2% in a sample of individuals with T1D aged 13–55 years (Deiana et al., 2016). Rates of IO among children and adolescents with T1D are currently somewhat unclear, perhaps partly because young people are often reluctant to disclose issues with their diabetes teams (Candler et al., 2018). However, the use of IO as a weight control has been reported in 2% of preadolescent girls (Colton et al., 2004), and 11–15% in adolescent girls (Jones et al., 2000). Furthermore, case studies of young people with T1D, girls in particular, are beginning to emerge (e.g. Kinik et al., 2017), suggesting that persistent IO for the purpose of weight loss is a prevalent behaviour among children and adolescents, as well as adults. A number of the risk factors of IO explored in this review are also seen as having greater prevalence among young people, suggesting that children and young people who have T1D, alongside one or more of these risk factors, will be at significant risk of engaging in IO as a weight loss method.

## Methods

The authors prepared this review from literature searches in Science Direct, PsychINFO, PubMed and Google Scholar. After reviewing the available literature relating to diabulimia, IO and T1D, we identified the following factors as posing the most significant risk to the development of IO-related behaviour: gender, the presence of anxiety and depression and weight concern and body dissatisfaction. Each of these risk factors will be explored in relation to the development of IO below.

## Gender

Gender is well referenced as a significant factor in the development of several mental health conditions, with women and young girls being more susceptible to developing conditions including depression, anxiety and eating disorders. Research illustrates a lifetime prevalence of generalised anxiety disorder of 7.7% among women, compared to 4.6% in men (Kessler et al., 2012). Similarly, rates of depression have been demonstrated as high as 21.3% among women, compared to 12.7% of men (Kessler et al., 1993). Among adolescents with T1D, research suggests that prevalence rates of depression are higher in girls than boys, and that depression in girls predicts poorer T1D treatment adherence (Korbel et al., 2007).

Gender differences also appear prevalent in eating disorders (EDs); overall prevalence rates among adolescents have been demonstrated at 20.8% in girls and 14.9% in boys (Sepulveda et al., 2008). Girls as young as 10 years old are also significantly more likely than boys to perceive themselves as being a larger size than they are (Thompson et al., 2003), more likely to report lower levels of body satisfaction (Strong et al., 2000), and more likely to display weight concern (Calzo et al., 2012).

Rates of disordered eating behaviours among females with T1D have been shown to range between 30 and 40% compared to between 9 and 11% in males (e.g. Baechle et al., 2014). Given IO’s utilisation as a weight loss technique, and drive for thinness being a critical element of ED diagnosis (Garner, 2004), it is logical that a number of research papers have highlighted that the number of females with T1D engaging in IO appears to be substantially higher than males. Neumark–Sztainer and colleagues (2002) found that among their sample, 10.3% of adolescent females admitted to IO (7.4% did so to lose weight), compared to just 1.4% of adolescent males. This is supported by Jancin (2010), who found a female to male ratio of IO of 10:1. However, a recent population-based study has suggested that this difference between genders may not be as great as earlier thought, with rates of female and male insulin restriction at 20.5% and 18.5%, respectively (Baechle et al., 2014). Unlike the 2002 study, which required participants to have been diagnosed with T1D for at least 1 year, Baechle and colleagues’ study included participants with early onset of T1D (diagnosis between 0 and 4 years of age). This variation in results could suggest that prevalence rates of IO between genders may differ depending on age of diagnosis, although further research is necessary to support this.

Interpreting these gender differences in rates of IO should be done with caution, as one study suggests that boys with body issues are more likely to overexercise as a weight loss method, rather than restrict their diet (Ricciardelli & McCabe, 2004). This would not always be implicated as disordered behaviour in T1D using traditional questionnaires like the DEPS-R, but could result in IO as a weight loss method. Furthermore, research indicates that male ED sufferers often do not seek out help until their illness becomes severe (Dearden & Mulgrew, 2013), and only 15% of men with an ED will seek treatment (Freeman, 2005), therefore skewing perceptions of gender bias in eating disorders and associated behaviours like IO. However, within a sample of both male and female participants who engaged in IO as a method of weight loss, females were found to have significantly higher HbA1c than males, indicating that they engaged in the behaviour more frequently or for more prolonged periods of time (Deiana et al., 2016), suggesting that females are at a higher risk of developing more significant long-term consequences as a result of IO.

## Anxiety and depression

A number of mental health conditions have been found to have higher prevalence among people with T1D compared to their peers, particularly anxiety and depression. Prevalence rates of anxiety are vastly higher in people with T1D compared to the general population; between 14–21% and 6%, respectively (Bernstein et al., 2013; McManus et al., 2016). While there is currently a few research looking at the association between anxiety and IO, higher levels of HbA1c have been found in people with T1D and comorbid anxiety (Shaban et al., 2006), which could indicate engagement in insulin omitting behaviours. Further research looking at this comorbidity is necessary to draw causality.

With regards to depression, a systematic review found an almost 4-fold increase of depression in T1D compared with controls (12.0% vs. 3.2%, respectively; Barnard et al., 2006). Due to the debilitating nature of depression, everyday behaviours like exercise, maintaining a healthy diet and managing physical illnesses can become more difficult (Lin et al., 2004). Comorbidity of depression and T1D has been found to associate with less adherence to treatment (Gonzalez et al., 2008) and higher HbA1c (van Tilburg et al., 2001). This comorbidity has recently been shown as bidirectional, where higher depressive feelings lead to poorer diabetes management, and poorer diabetes management causes increased depression (Nouwen et al., 2019). While there is limiting causal research implying the direct effect of depression on engagement in IO, one study of 9–13-year-old girls (Olmsted et al., 2008) highlighted that those who admitted to IO scored positively on the Children’s Depression Inventory (Kovacs, 1992). However, the issue of causality is difficult. Even in children and adolescents, depression has demonstrated distinct comorbidity with EDs (Swanson et al., 2011), and levels of depressive symptoms have been shown to improve following weight gain in hospitalised eating disorder patients (Sala et al., 2011). Depressive symptoms may also be experienced in patients engaging in IO, given that resulting hyperglycaemia is associated with low energy levels, poor sleep and trouble concentrating ((Ruth-Saad et al., 2009). Future research is necessary to further explore the causal relationship of depression on IO.

## Increased weight concern and body dissatisfaction

Weight concern and body image issues are common among women (Wardle & Johnson, 2002) and increasingly recognised among men (Thompson, 2017); this is often evident from childhood and adolescence. A study of 1515 children aged 9–14 years of age reported high levels of body dissatisfaction: 50.5% of girls and 35.9% of boys wanted a thinner body shape, while 7.2% of girls and 21.1% of boys wanted a larger shape (Dion et al., 2016).

The early emergence of body dissatisfaction has unsurprisingly been linked to the development of EDs, including anorexia nervosa (Button & Whitehouse, 1981) and bulimia nervosa (Watson et al., 2011), in childhood and adolescence. The frequency of EDs varies widely across age and gender, but Stice and Bohon (2012) report an overall lifetime prevalence of anorexia between 0.9 and 2.0% for women and 0.1 and 0.3% for men, and a lifetime prevalence of bulimia between 0.2 and 3.5% for women and 0.9 and 2.0% for men. Evidence of increased prevalence among children is also beginning to emerge, with rates of bulimia among youth as high as 2% (Merikangas et al., 2010).

In addition to the common factors listed above, patients with T1D face two additional challenges, and may experience negative associations with their body shapes as early as diagnosis. Firstly, prior to diagnosis many individuals with T1D experience substantial weight loss due to the hyperglycaemic state caused by insufficient insulin production. Once diagnosed and upon the commencement of an insulin regime, patients will start gaining weight as their body regains the fluids it needs and rebuilds its fat stores. This weight gain sometimes exceeds pre-T1D levels, and a high proportion of individuals become overweight or obese (Newfield et al., 2009). This noticeable, and sometimes sudden, weight change can cause serious concern in patients with T1D and is often attributed to the use of insulin (Larger, 2005). With the average age of diagnosis being between 10 and 14 years old (Diabetes UK, 2010), children and adolescents with T1D may begin to experience body shape and weight issues earlier compared to their peers.

Secondly, following diagnosis, weight is regularly monitored and reviewed in the management of T1D. Body mass index is one of eight care process checks recommended by the National Institute for Health and Care Excellence, 2015, meaning patients are routinely weighed during their regular medical check-ups. The constant weight checks, along with the forced dietary monitoring required to adequately control blood glucose, can create additional negative associations between body size and eating behaviours in patients with T1D. This in turn may also lead to the development of further weight concerns, body dissatisfaction and eating disorder–related behaviours. Cumulatively, these factors are surmised to be influential in the development of EDs.

There may be merit in changing diabetes clinical practice to reduce these two additional challenges that children and adolescents with T1D face. For example, young patients and their families could be reassured by their diabetes team about the way the human body temporarily overshoots after a period of hyperglycaemic (starvation) state, such as that which occurs at the onset of diabetes. In addition, clinicians could explain expected weight and height increases during normal growth and puberty and can encourage more positive blood glucose management methods, such as moderate physical activity and dietary plans, that do not place so much emphasis on patients’ weight itself. Providing the patient and their family with sufficient education at the point of diagnosis is the key to success (Acerini et al., 2014).

A meta-analysis suggests that individuals with T1D are up to three times more likely to suffer with an ED than their peers (Young et al., 2013). Furthermore, cross-sectional research suggests that rates of insulin misuse is increased among patients with T1D where an ED also coexists (Nielsen, 2002), suggesting a close link between T1D mismanagements and disordered eating behaviours. Peveler et al. (2005) reported that weight concern and body dissatisfaction also play an important role in the development of insulin misuse in children and adolescents, as well as in adults. While not all incidences of IO are related to an eating disorder diagnosis (see ‘Other Functions of IO’), rates of IO among individuals with T1D and comorbid ED are significantly elevated, therefore suggesting the significance of weight concern and body dissatisfaction as risk factors in the development of IO.

### Complications associated with prolonged IO

Insulin omission causes hyperglycaemia, which can lead to several vascular complications associated with increased morbidity and mortality (Goebel-Fabbri et al., 2008). Understanding the risk factors associated with developing the behaviour is therefore crucial in reducing the likelihood of developing the below complications.

### Retinopathy

Prolonged hyperglycaemia can lead to the formation of microaneurysms in the retina, which can cause occluded vision and eventually lead to blindness (Donaghue et al., 2018). This is associated with significant reduction in quality of life (Brown et al., 2002) and increased mortality (Kramer et al., 2011). Nielsen (2002) found that retinopathy took, on average, just 3.4 years to develop in patients who engaged in IO, compared to 11.5 years in those with T1D but without IO.

### Neuropathy

Persistent hyperglycaemia can lead to nerve damage, which reduces vascular flow and can result in amputation (Ziegler et al., 1988). The 3-year survival rate in diabetic patients with neuropathy complications has been found at less than 17% (Ramsey et al., 1999). Cases of neuropathy are also disproportionately found in those who omit insulin; Steel and colleagues (1987) found that of the nine participants in their sample who admitted to IO, 5 (55.5%) displayed symptoms of neuropathy.

### Nephropathy

Lengthy periods of hyperglycaemia can result in structural damage to the kidneys, causing reduced function and eventual kidney failure (Donaghue et al., 2018). Diabetic nephropathy is generally accepted as the leading cause of mortality among patients with T1D, with lifetime incidence rates of approximately 50% (Marshall, 2012). This is particularly significant, given recent longitudinal evidence illustrating a mortality rate due to nephropathy of 32.8% (Ang et al., 2014).

### Cerebral oedema

The rapid change of extracellular brain fluid caused by rapid correction of hyperglycaemia can lead to significant complications (Varela et al., 2018). Cerebral oedema is recognised as one of the most dangerous complications of hyperglycaemia, with a mortality risk of 20–25%; it is also considered to account for 60–90% of deaths during diabetic ketoacidosis (Rosenbloom, 2010; Wolfsdorf et al., 2006).

## Diabetic ketoacidosis and mortality

Severe dehydration and loss of electrolytes caused by acidosis can quickly lead to coma and death. The mortality rate associated with diabetic ketoacidosis in hospital admissions has been displayed at 13% (Efstathiou et al., 2002). Engaging in IO increases the risk of developing all of these complications associated with hyperglycaemia, which in turn can lead to early mortality.

### Other functions of IO

While the function of IO has been found to be for weight loss purposes in around half of instances (Polonsky et al., 1994), it is important to acknowledge other functions of the behaviour. One example is the use of IO as a form of self-harm. ‘Taking too little medication’ is a method of self-harm listed the Ottawa Self-Injury Index (Nixon et al., 2002), and given the resulting negative physiological impact which results from IO, the behaviour could be classed as a form of self-injury. Support for this was found in one study where self-destructive behaviour was stated as the cause of 28% of IO cases, compared to weight loss which only accounted for 15.5% (Schober et al., 2011). While the available literature is still limited, there is more evidence emerging illustrating the use of IO as a form of deliberate self-harm (e.g. Staite et al., 2018).

Other prevalent causes of IO include injection anxiety, particularly in children (Young-Hyman et al., 2016), and fear of hypoglycaemia (low blood sugar; Wild et al., 2007). Diabetes burnout, a state of exhaustion towards the condition associated with lack of treatment adherence and deficient blood glucose control (Young-Hyman et al., 2016), may also result in omission of insulin. In this instance, people with T1D report feeling both physically and mentally tired of the constant need for self-care (Abdoli et al., 2020) and are therefore unable to manage their diabetes control as adequately as is necessary. If there is no associated desire to lose weight, then diabetes burnout could be considered an alternative function of IO.

Given the multifaceted nature of T1D and its associated physiological and psychological impact, the function of IO will vary widely and may serve more than one purpose. Therefore, as clinicians become more aware of IO, it is important to consider a number of factors before determining its function; taking into account the complex nature of IO may mean that its functions could change over time.

## Conclusions

This literature review illustrates that gender, anxiety and depression and increased weight concern and body dissatisfaction may all represent significant risk factors in the development of IO. As has been highlighted, a number of these factors have also been demonstrated as having distinct comorbidities. Comorbidity of two or more of these factors may therefore present an even greater risk of engaging in IO behaviours, although there is currently little research to support this.

Insulin omission is an extremely complex behaviour, and while the list of factors presented in this article were selected based on the research evidence suggesting their association with the development of the behaviour, it does not attempt to be exhaustive. Several further factors have been identified within the literature as risk factors to the development of the behaviour. These include family conflict (Vaid et al., 2018), media influence (Hackman, 2015), socioeconomic status (Hassan et al., 2006) and self-regulatory capacity (Lansing & Berg, 2014). Researchers should take such factors into account before drawing conclusions based on their data.

The importance of expanding this research field cannot be understated, given the dangerous complications associated with the prolonged hyperglycaemia caused by IO, and the research indicating that it is a behaviour which not only affects adults but children and adolescents as well. Further research may wish to consider the comorbid relationships between the risk factors presented in this article, to develop a clearer understanding of how and to what extent each of these factors increase the risk of engaging in IO among children and adolescents with T1D.
